# Implementing and testing Bayesian and maximum-likelihood supertree methods in phylogenetics

**DOI:** 10.1098/rsos.140436

**Published:** 2015-08-05

**Authors:** Wasiu A. Akanni, Mark Wilkinson, Christopher J. Creevey, Peter G. Foster, Davide Pisani

**Affiliations:** 1 Department of Biology, The National University of Ireland, Maynooth, Co. Kildare, Republic of Ireland; 2 Institute of Biological, Environmental and Rural Sciences (IBERS), Aberystwyth University, Aberystwyth, Ceredigion SY23 3FG, UK; 3 Department of Life Science, The Natural History Museum, London SW7 5BD, UK; 4 School of Biological Sciences and School of Earth Sciences, University of Bristol, Life Sciences Building, 24 Tyndall Avenue, Bristol BS8 1TG, UK

**Keywords:** phylogeny, Bayes, maximum likelihood, support

## Abstract

Since their advent, supertrees have been increasingly used in large-scale evolutionary studies requiring a phylogenetic framework and substantial efforts have been devoted to developing a wide variety of supertree methods (SMs). Recent advances in supertree theory have allowed the implementation of maximum likelihood (ML) and Bayesian SMs, based on using an exponential distribution to model incongruence between input trees and the supertree. Such approaches are expected to have advantages over commonly used non-parametric SMs, e.g. matrix representation with parsimony (MRP). We investigated new implementations of ML and Bayesian SMs and compared these with some currently available alternative approaches. Comparisons include hypothetical examples previously used to investigate biases of SMs with respect to input tree shape and size, and empirical studies based either on trees harvested from the literature or on trees inferred from phylogenomic scale data. Our results provide no evidence of size or shape biases and demonstrate that the Bayesian method is a viable alternative to MRP and other non-parametric methods. Computation of input tree likelihoods allows the adoption of standard tests of tree topologies (e.g. the approximately unbiased test). The Bayesian approach is particularly useful in providing support values for supertree clades in the form of posterior probabilities.

## 1. Introduction

Supertrees [1] are phylogenies that are built by combining the information contained in a set of input trees. Different supertree methods (SMs) offer alternative ways to amalgamate input tree information. Wilkinson *et al.* [2] distinguished between liberal SMs, which may be able to resolve conflicts among input trees, and conservative SMs (e.g. [3]) that cannot do so. Here, we are concerned only with liberal SMs. These include, for example, various forms of matrix representations with parsimony (MRP) [4,5] or compatibility [6–8], the average consensus [9], the most similar supertree (MSS) [10]; minimum flip [11], and minimum cut [12,13] methods. Most liberal SMs are somewhat ad hoc and many lack seemingly desirable properties or have seemingly undesirable ones [2,14] including biases with respect to input tree size [6] and shape [15]. Nonetheless, these SMs, especially MRP, have been used to build supertrees for many animal, plant and microbial taxa (e.g. [16–18]) either from partially overlapping phylogenies extracted from the literature (e.g. [8,19,20]) or from trees generated de novo including large sets of gene trees in phylogenomic studies (e.g. [10,21,22]).

The relatively recently defined majority-rule, MR(-) SM [23,24], which is a generalization of the well-known and well-founded consensus method of the same name [25], is a liberal SM that is not known to suffer from input tree size or shape biases and that has a number of other seemingly desirable properties that most currently available SMs lack. MR consensus trees can be defined as the strict consensus of the trees that minimize the sum of their distances to the input trees where distances between trees are given by the Robinson & Fould [26] metric (RF—i.e. the symmetric difference on full splits). The corresponding MR(-) SM is based on a generalization of the RF distance to the case where input trees have different leaf sets. Note that the RF SM [27] is similarly based on minimizing the RF distance but is slightly different in returning only fully resolved supertrees and has been suggested to represent a useful starting point in the estimate of the MR(-) supertree [27].

Steel & Rodrigo [28] introduced a different (though closely related, see below), parametrized approach to supertree construction based on the ideas that the input trees can be considered a sample of subtrees drawn from some unknown supertree and that we can model the probability distribution of topological error (i.e. incongruence with the supertree). They developed a simple general model (which we shall call SR 2008) of input tree–supertree incongruence as an exponential probability distribution of some measure of input tree to supertree distance, and a more specific model (SR–RF 2008) that, like the MR(-) SM, uses RF distances as the input tree to supertree distance. These models allow likelihoods of candidate supertrees to be defined and thus for maximum-likelihood (ML) supertrees to be sought. By extension they also pave the way for a corresponding Bayesian Markov chain Monte Carlo (MCMC) approach to supertree construction.

According to SR [28], unlike MRP, the ML SM should be statistically consistent under fairly general conditions. Parametric SMs have other attractions including the possibility of applying model choice and hypothesis testing approaches that are routine in other areas of phylogenetics but hitherto lacking in supertree construction and which should facilitate the entry of supertree construction into the mainstream of phylogenetics [29]. In addition, extending the use of these models into a Bayesian framework would offer bootstrap-independent support measures (i.e. posterior probabilities) that circumvent difficulties that are frequently associated with bootstrapping in the supertree context [17,30–34] especially where input trees are harvested from the literature.

We recently described the first software implementation of the ML SM using the SR–RF 2008 model [35]. Here, we demonstrate this, and a heuristic implementation of a corresponding Bayesian SM and we compare the performance of these new parametric methods with that of the MR(-) and some other current liberal SMs using real and hypothetical examples. An alternative Bayesian supertree approach, based on the analyses of a standard matrix representation of a set of input trees, was introduced by Ronquist [36] but has remained unused and thus we do not consider it further here.

## 2. Material and methods

### 2.1 Background theory

We wish to estimate a supertree that displays inferred phylogenetic relationships among all the leaves from a set of input trees each of which may include only a proper subset of the leaves and which may be incongruent (for a variety of reasons) with the supertree. In the SR–RF 2008 model, incongruence between candidate supertrees and input trees is measured with RF distances and, for a collection of input trees, is assumed to follow an exponential distribution such that the farther any given input tree is from the underlying true supertree the lower its probability [28]. To calculate the RF distance between a supertree and an input tree that contains fewer species the supertree is restricted (by pruning leaves) to the leaf set of the input tree [23]. Equation (1) (SR's [28] eqn 1) gives the probability of an input tree T’ on leaf set Y, given the supertree T, where *α* is a normalizing constant intended to ensure that the sum of the likelihoods of all the supertrees is equal to one, *β* is a parameter that is free to vary in relation to both the quantity and the quality of the data and *δ* is a measure of the distance (in our case the RF distance) between T and T’. The ML supertree is that which maximizes the probability of the set of input trees (for further details, see [28]).

2.1 $$P_{T,Y}[T^{\prime }]=\alpha \exp[-\beta \partial (T^{\prime },T|Y)].$$

SR [28] initially claimed that *α* depends only on *β* and the size of T’, making it easy to calculate. However, Bryant & Steel [37] subsequently showed that *α* depends also upon input tree shape, such that calculating *α*, although possible in polynomial time, is extremely computationally expensive when it has to be calculated for every proposed supertree. Bryant & Steel [37] suggested that *α* could be ignored if a sufficiently low or high *β* is used, because the ranking of the supertrees will be unaffected.

We developed a heuristic implementation [35] of SR–RF 2008 [28] ML SM, where *α* is ignored and the *β* values are kept within the ranges that are considered to be unproblematic by Bryant & Steel [37]. Our current implementation of the ML SM uses subtree pruning and regrafting to explore tree space and is not particularly efficient, restricting its use to relatively small datasets. However, it allows the likelihood of any supertree to be estimated (given a set of input trees) and compared, and the implementation of tests of two trees (including the approximate unbiased test [38]) in the supertree context.

Being able to estimate the likelihood of a supertree given a set of input trees also allows the implementation of a corresponding Bayesian MCMC approach. Bootstrapping can be more or less problematic in the supertree context [17,31,32,39] depending on the effectiveness of overlap among the input trees. Thus, Bayesian SMs have the substantial potential advantage of being able to provide bootstrap-independent measures of branch support in the form of posterior probabilities. Based on the results of SR [28] and Bryant & Steel [37], the first Bayesian SM using the SR–RF 2008 model was implemented as part of the phylogenetic software p4 [40]. The Bayesian supertree approach is faster than our ML implementation and allows for the analysis of phylogenomic scale datasets. The current implementation is restricted to the use of fully resolved input trees.

### 2.2 Tests and comparisons

We tested our implementations of the model-based SMs for potential biases with respect to input tree size and shape using the examples of Purvis [6] and of Wilkinson *et al.* [15], respectively. We ran ML analyses in L.U.st [35] using the default heuristic tree search option for 10 iterations. Except where stated otherwise, all analyses performed with the Bayesian SM were executed by running two MCMC chains with *β*=1.

ML supertrees under the SM–RF 2008 model are closely related to the MR(-) SM [28] (see below) and we compared these two SMs together with MRP, MSS, RF and our Bayesian implementation using the small (five gene trees, a total of nine taxa) *Drosophila* dataset of Cotton & Wilkinson [23] for which the MR(-) supertree is already known. The Bayesian analysis was run for 1000 iterations, sampling every 20 generations.

Effective tree search for large datasets is not possible using our current implementation of the ML SM but is more tractable with the corresponding Bayesian implementation. We conducted Bayesian analyses of two larger scale empirical datasets, one genomic and the other based on trees sourced from the literature. Firstly, we analysed the metazoan dataset of Holton & Pisani [22], which includes input trees based on 2216 gene families from a total of 42 species, running MCMC chains for 10 000 iterations and sampling every 100 generations while employing various values of *β* (0.001, 0.01, 0.1 and 0.5). Differences in the results were minimal and only those obtained with *β*=0.5 values are reported. This represents a genomic scale application of supertrees and because Holton & Pisani [22] previously analysed this dataset using MRP and were able to estimate support for the nodes in this tree using input tree bootstrapping it is a useful dataset for comparing bootstrap support and posterior probabilities.

Secondly, we conducted a de novo analysis using a carnivore dataset based on that of Nyakatura & Bininda-Emonds [18], which originally comprised 558 input trees sourced from the literature (including polytomous trees and their various resolution) for a total of 286 taxa. We modified this dataset as follows. Firstly, we generated 10 datasets each containing just one randomly resolved tree for each polytomous input tree from the original dataset. Secondly, we did not include the taxonomy tree and excluded taxa present only in the taxonomy tree. Also unlike the original analyses, we did not apply any differential weighting of input trees or input tree clades. Each of the 10 datasets, comprising 274 fully resolved input trees for a total of 271 leaves, was analysed by running MCMC chains for five million iterations, sampling once in 1000 generations. The trees sampled after convergence from all 10 runs (a total of 30 020) were then merged, using the MR consensus, to generate a single Bayesian supertree. We compared the Bayesian supertree with both the original MRP tree of Nyakatura & Bininda-Emonds [18] and with a new MRP supertree (generated on the same dataset we used for the Bayesian analysis, i.e. without differential weighting of input trees or clades and without a taxonomy tree). For the latter, we used the phylogenetic package CLANN to generate the MRP matrix and PAUP4b10 [41] to analyse the data, saving trees from 100 heuristic searches (with random sequence addition and tree bisection reconnection) with the multrees option turned off and using these as starting trees for an analysis with the multrees option turned on (and tree bisection reconnection) and using an MR consensus to summarize the resulting set of 585 166 most parsimonious supertrees (see also [20]).

From each of the 10 datasets analysed using the Bayesian approach, a set of 100 supertrees saved (after convergence) were sub-sampled. These 1000 trees were used as inputs to a subsequent leaf stability analysis [42] performed in p4, to investigate the presence of rogue taxa (*sensu* [43]). The 26 taxa registering a score above the average score for the entire dataset (i.e. relatively unstable taxa) according to the leaf stability analysis were deleted from the set of 30 020 Bayesian supertrees and from the 585 166 most parsimonious trees obtained from the MRP analysis (keeping duplicate trees), and new (MR consensus) supertrees constructed. Finally, to allow for a full comparison across all the considered trees, these 26 relatively unstable taxa were also removed from the original carnivore MRP supertree presented by Nyakatura & Bininda-Emonds [18].

Following Ronquist *et al.* [44], assessment of the convergence between the pairs of Bayesian MCMC chains was measured in p4 by calculating the average standard deviation of the splits frequency (ASDSF) of the sampled trees [44] with an ASDSF value of 0.01 or less considered indicative of convergence. A burn-in of 500 was used for all analyses.

For comparison, the carnivore and metazoan data were also analysed with the MSS SM implemented in CLANN [33] using the default options, and with the RF SM using the software of Bansal *et al.* [27] with the default parameters. The approximately unbiased (AU) [38] test as implemented in L.U.St [35] was used to compare alternative supertrees (Bayesian, MRP, RF, MSS). In the context of supertree reconstruction, the AU and other similar tests of trees are implemented by calculating input tree specific likelihood values for each of the compared supertrees. This is akin to estimating site-specific likelihood values in the case of the standard, sequence based versions of these tests. Once these input tree specific likelihood values are available, they can be used in consel [45] to calculate the AU and other tests. Samples of 100 random (super)trees were generated (using PAUP4b10), their likelihoods estimated (using the L.U.st package [35]) and compared to those for the various inferred supertrees, to better understand to what extent these methods perform better than random.

## 3. Results

Purvis [6] used two conflicting input trees of different sizes to show that MRP was biased in relation to the size of the input trees in how it resolves conflict and that this was caused by how input trees are represented in the matrix. Similarly, Wilkinson *et al.* [15] used two conflicting input trees of different shapes to show that MRP and other liberal SMs suffer from input tree shape biases due to objective functions based on unusual asymmetric measures. Based on the analyses of these hypothetical examples, the parametric SMs show no evidence of any obvious bias with respect to input tree size or shape: the ML analyses returned sets of supertrees for which the strict consensus trees were completely unresolved and the Bayesian analyses failed to converge.

Applied to the *Drosophila* dataset, ML recovered the same set of 79 supertrees that Cotton & Wilkinson [23] found which minimize the sum of the supertree–input tree RF distances, the strict consensus of which is the MR(-) supertree. Bayesian analysis produced a tree topologically identical to this strict consensus. The MRP analysis of the same dataset returned 77 equally parsimonious trees, MSS found 42 supertrees, RF found 26 supertrees and in each case the supertrees returned are a subset of the 79 supertrees that minimize the sum of the supertree–input tree RF distances and their strict consensus is identical to the MR(-) supertree. There is substantial but incomplete correspondence between the likelihood and parsimony fits of the input tree dataset to all possible supertrees (not shown).

**Figure 1.**
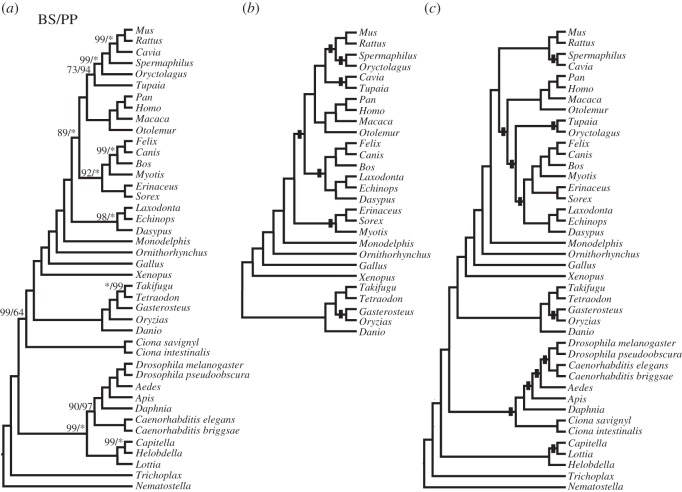
Phylogenomic supertrees of the metazoan based on the dataset of Holton & Pisani [22]. (*a*) Bayesian supertree with posterior probabilities (PP) along with the bootstrap support (BS) values obtained by Holton & Pisani for the MRP tree. Asterisks and clades with no values shown have maximum support. (*b*) The MSS (only the part of the tree that conflicts with the Bayesian tree is shown). (*c*) Strict consensus of the 15 supertrees inferred with the RF SM. The black square indicates clades that are biologically implausible. This dataset is composed of 2216 gene trees overlapping on 42 taxa. Taxa names are shortened to their genus names to make figure more readable.

**Figure 2.**
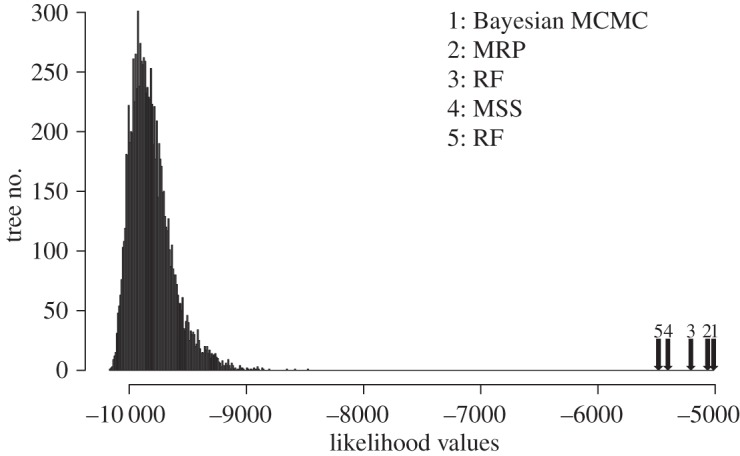
A graph showing the comparison of the distribution of the likelihood scores for 1000 random supertrees on the same taxon set of the metazoan dataset and the likelihood scores for the metazoan phylogeny inferred by the MSS, RF, MRP and Bayesian SMs.

The Bayesian (MCMC) analysis of the metazoan dataset converged after 2500 iterations. Figure 1*a* is the MR consensus of 150 supertrees sampled after convergence, summarizing relationships and support (estimated posterior probabilities of full splits) and is topologically identical to the MRP tree of Holton & Pisani [22]. Posterior probabilities for the nodes in this tree are also very similar to the bootstrap support values obtained in the original MRP analysis [22]. Quite importantly, the relationships in this tree are in full agreement with current understanding of animal relationships. This is not the case for supertrees inferred using MSS (figure 1*b*) and RF (figure 1*c*). The MSS incorrectly resolves the relationships among the mammal species, while the RF supertree has many obviously incorrect clades, such as chordates (*Ciona*) forming a sister group relationship with arthropods (*Daphnia*, *Drosophila*, *Apis*, etc.). Likelihoods of each of these supertrees are compared to each other and to the distribution of likelihoods for a set of 1000 random supertrees in figure 2. Although all the inferred supertrees fit the data better than the random trees, AU tests indicate that the RF supertree (the worst of those considered judged in terms of their likelihoods) and MSS have significantly worse fits to the data (*p*=0.001 and *p*<0.00001, respectively) under the SR–RF 2008 model than do the MRP and Bayesian supertrees.

**Figure 3.**
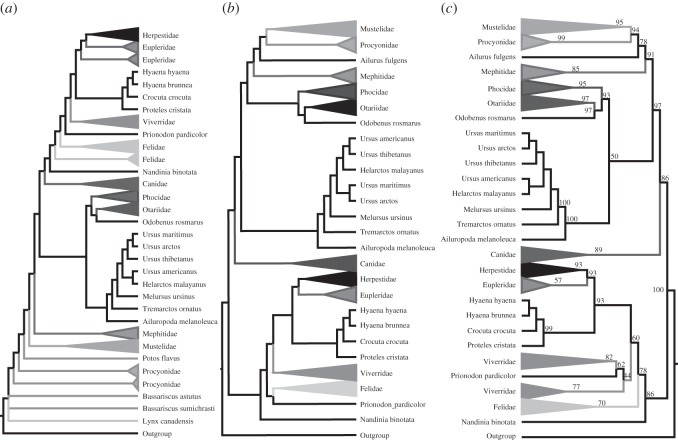
Phylogenomic supertrees of the Carnivora. These supertrees have been reconstructed from 274 gene trees overlapping on 245 taxa and had 26 unstable taxa (according to a leaf stability test) pruned. (*a*) Phylogeny inferred by MRP with equal weighting of clades. (*b*) Phylogeny inferred by differentially weighted MRP with a taxonomy tree [18]. (*c*) Phylogeny inferred by the Bayesian MCMC SM with posterior probabilities shown.

**Figure 4.**
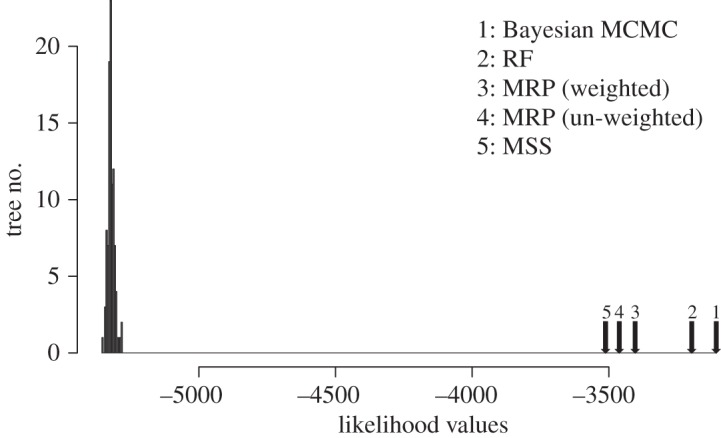
A graph showing the comparison of the distribution of the likelihood scores for 100 random supertrees on the same taxon set as the phylogenies in figure 3 and the likelihood scores for the Carnivora phylogeny inferred by the MSS, RF, MRP (differentially weighted and equally weighted) and Bayesian SM.

With the carnivore example, the Bayesian supertree (after pruning unstable taxa) has generally high levels of support (figure 3*a*) and is in good agreement with the original MRP supertree [18]. At the ordinal level, the only differences are two clades that have low support in the Bayesian tree, suggesting that there is not much signal in the data and that the alternative placements of these taxa cannot be considered reliable. By contrast, the newly generated MRP tree (figure 3*b*), constructed using the same dataset as that used for the Bayesian supertree analysis (i.e. without a taxonomy tree and with equal weighting of input trees), is very different and biologically highly implausible in several respects. Supertrees built with the MSS and RF SMs (not shown) differed to some extent from the Bayesian supertree. The AU test shows that the Bayesian supertree fits the data significantly better than the original MRP supertree (*p*=0.003), the MSS (*p*=0.002) and our newly generated MRP tree (*p*=10^−49^) under the SR–RF 2009 model. Only the RF supertree did not have a significantly worse fit to the data (*p*=0.343). Figure 4 shows that all the considered methods returned supertrees that, judged by their likelihoods, are significantly better than expected for randomly selected trees.

## 4. Discussion

Model-based, statistical inference has been widely adopted in phylogenetics. However, until very recently, the many advantages that such approaches can offer have not been available within the field of supertree construction which has been dominated by the use of non-parametric methods, particularly MRP. SR's development of a simple mathematical model of the distribution of topological error in a set of subtrees, sampled with some distortion (incongruence) from a supertree [28], has provided a crucial first step in extending the tools and techniques of ML and Bayesian inference into the domain of supertree construction and evaluation. Our implementations and tests of ML and Bayesian supertrees based on the SR–RF 2008 model provide a further proof of concept for the applicability of these methods. Our theoretical and empirical examples indicate that the statistical methods perform no worse, and in some cases clearly better, than more ad hoc methods. Our results show that for the metazoan dataset, the Bayesian SM returns trees that fit the data as well as the MRP tree, while for the more challenging carnivore dataset, our Bayesian SM returns trees that fit the data substantially better than the MRP tree. Additionally and importantly these parametric methods allow statistical tests of alternative trees and the Bayesian version provides measures of clade support in terms of posterior probabilities.

SR [28] considered their RF-based ML SM to be equivalent to the MR(-) SM of Cotton & Wilkinson [23], but this supposed equivalence was based on the mistaken idea that the *α* parameter was determined by the *β* parameter and the size but not the shape of the input trees [37]. Consequently, while these methods are expected to be similar they are not identical. Further, the dependence of the *α* parameter upon input tree shape makes its calculation much more complicated. Bryant & Steel [37] suggest conditions under which this complication can be ignored without impacting upon the rankings of supertrees based on their approximated likelihood scores and we have operated within these conditions in our analyses using this method. However, Bryant & Steel [37] caution that their findings may not be of much help in a Bayesian setting. Thus, we remain concerned to emphasize that current Bayesian implementations must be considered doubly heuristic in the search of tree space and in the approximation of the likelihoods and that further work is needed on the impact of such approximation.

Irrespective of these approximations in all performed tests the Bayesian and the ML supertree approaches fared as well as or better than any of the compared methods. It is noteworthy that while the Bayesian SM did as well as MRP in the case of the Metazoa dataset (where taxon overlap is high among the input trees), this method outperformed MRP in the case of the revised carnivore dataset, which represents a much more difficult example (because of the smaller taxon overlap between input trees). Indeed, whereas the Bayesian supertree (after pruning rogue taxa) is essentially consistent with the original MRP supertree obtained using differential weighting and a taxonomy tree, the de novo MRP analysis without differential weighting and without a taxonomy tree was quite unsuccessful.

The development of model-based phylogenetics has seen the earliest, simplest and most mathematically tractable models of sequence evolution supplemented with ever more complex and more realistic, parameter rich models, together with techniques for choosing among alternative models and for assessing model adequacy. We anticipate a similar future for model-based supertree construction in which the simple SR–RF 2008 will be supplemented by other models that use different measures and assume distributions of topological error with standard methods of choosing among alternative models. Further we anticipate the use of mixture models to provide *a posteriori* classifications of the various causes of incongruence among sets of gene trees in phylogenomic scale studies. SMs are only one of several approaches to phylogenetic inference from diverse datasets that include also concatenation and gene tree-species tree reconciliation methods. Comparison of SMs with these other approaches is desirable but beyond the scope of this work.

## 5. Conclusion

Supertree reconstruction is coming of age [29]: we can now recover supertrees using methods with interesting and well-understood mathematical properties (e.g. that of being median trees for the input set), that have a strong and clear mathematical underpinning (i.e. they are not black boxes), and that are not expected to recover clades on the ground of biases or other unwanted properties. In addition, support for the nodes on these supertrees can now be easily estimated.

Supertrees have previously found interesting implementations in phylogenomics and to generate phylogenetic frameworks to be used in macro-evolutionary studies. In both cases, however, the use of supertrees was hampered by worries about the nature of the relationships displayed on the tree, and by difficulties in estimating support for the relationships reported. The availability of the Bayesian and ML methods eliminates both of these issues and it should now be expected that the use of SMs with undesirable properties (e.g. MRP) should dwindle, and that supertrees would become at the same time more broadly used. Some problems of SMs still exist. For example, how we optimally deal with data redundancy when supertrees are used as in Nykatura & Bininda-Emonds [18] to summarize trees from the literature is still an open question. However, studies have been performed and weighting schemes proposed that can take care of these problems, and improvements of these weighting schemes can be expected. In addition, these weighting schemes can easily be extended to the Bayesian and ML framework and it can be expected that their implementation will allow for further phylogenetic improvements.

## Data accessibility

The datasets supporting this article have been uploaded to Dryad with the following identifier: http://dx.doi.org/10.5061/dryad.h9541.
